# Monitoring Ion Track Formation Using In Situ RBS/c, ToF-ERDA, and HR-PIXE

**DOI:** 10.3390/ma10091041

**Published:** 2017-09-06

**Authors:** Marko Karlušić, Stjepko Fazinić, Zdravko Siketić, Tonči Tadić, Donny Domagoj Cosic, Iva Božičević-Mihalić, Ivana Zamboni, Milko Jakšić, Marika Schleberger

**Affiliations:** 1 Ruđer Bošković Institute, Bijenička cesta 54, 10000 Zagreb, Croatia; stjepko.fazinic@irb.hr (S.F.); zdravko.siketic@irb.hr (Z.S.); tonci.tadic@irb.hr (T.T.); donny.domagoj.cosic@irb.hr (D.D.C.); iva.bozicevic.mihalic@irb.hr (I.B.-M.); ivana.zamboni@irb.hr (I.Z.); milko.jaksic@irb.hr (M.J.); 2 Fakultät für Physik and CENIDE, Universität Duisburg-Essen, D-47048 Duisburg, Germany; marika.schleberger@uni-due.de

**Keywords:** swift heavy ion, ion track, RBS/c, AFM, ERDA, PIXE

## Abstract

The aim of this work is to investigate the feasibility of ion beam analysis techniques for monitoring swift heavy ion track formation. First, the use of the in situ Rutherford backscattering spectrometry in channeling mode to observe damage build-up in quartz SiO_2_ after MeV heavy ion irradiation is demonstrated. Second, new results of the in situ grazing incidence time-of-flight elastic recoil detection analysis used for monitoring the surface elemental composition during ion tracks formation in various materials are presented. Ion tracks were found on SrTiO_3_, quartz SiO_2_, a-SiO_2_, and muscovite mica surfaces by atomic force microscopy, but in contrast to our previous studies on GaN and TiO_2_, surface stoichiometry remained unchanged. Third, the usability of high resolution particle induced X-ray spectroscopy for observation of electronic dynamics during early stages of ion track formation is shown.

## 1. Introduction

Passage of swift heavy ions (SHIs) through a material may result in the formation of permanent damage along their trajectories called ion tracks. These nanoscopic objects are formed by a number of different physical processes that span femtosecond to nanosecond time domains. Ion tracks have been investigated for many years, and the development of this research field has been well documented [1,2,3,4]. Still, many questions related to the basic understanding of ion track formation are open and subject to vigorous scientific investigations [3,5,6,7,8,9,10]. Clarification of these intensely debated issues would benefit not only basic research but ion track applications as well [11,12].

Historically, ion track research has been spearheaded by research groups using large accelerator facilities. The reason for this is related to the existence of a threshold for ion track formation. Ion tracks can be formed only if the density of the deposited energy exceeds a certain critical value. According to the thermal spike models, a phase transition (most often melting of the material) that has to be triggered by a sufficiently high density of deposited energy is required for ion track formation. Only then does the relaxation of the deposited energy result in the formation of permanent damage, which is otherwise dissipated away without noticeable damage to the material. Various materials have widely different thresholds for ion track formation, and quite often high threshold values require SHI with kinetic energies in the 100–1000 MeV range for systematic investigations. However, ion track research now also takes place at medium accelerator facilities where kinetic energies in the range of ~10 MeV are available. At these energies, electronic stopping is still the dominant mechanism of the SHI kinetic energy deposition. The capability for ion track production given by the electronic stopping power of the SHI is a highly nonlinear function of its kinetic energy, and, therefore, medium accelerator facilities can deliver SHI beams of interest for ion track studies in many materials [13,14,15,16]. Often it is possible to pinpoint exactly the threshold for ion track formation. Practical considerations like limited availability of the beamtime can also play a significant role at large facilities, thus, it is advantageous for complementary investigations at lower energies to be outsourced elsewhere, as documented in our own research [10,17,18,19,20,21,22]. SHI applications can benefit from this also, for example, hadron therapy using carbon ions typically requires ~100 MeV beams in order to reach the targeted volume. However, the electronic stopping power maximum for carbon ions in water is at 5 MeV [23], thus, basic research related to understanding hadron therapy can be easily done at much lower energies.

In this work, we aim to present another important aspect of ion track studies at medium size accelerator facilities, namely, access to ion beam analysis (IBA) techniques that are practically not available at large accelerator facilities. IBA techniques are a set of powerful and versatile material science techniques that can provide elemental depth profiles and high resolution elemental or density distribution maps when performed on ion microprobes. For example, we have recently demonstrated imaging of etched ion tracks by scanning transmission ion microscopy (STIM) [15] and biological microstructures by MeV secondary ion mass spectrometry (MeV SIMS) [24]. In addition, ion microprobes can be used as a tool for nanoscale material patterning, like production of ordered arrays of etched single ion tracks [25] and MeV ion lithography [26].

A few of the IBA techniques, such as Rutherford backscattering spectrometry in channeling mode (RBS/c), ion beam induced charge (IBIC), and ionoluminescence (IL), can further provide information about defects in monocrystalline samples. Actually, RBS/c is one of the most often used techniques for ion track measurements because it is very suitable for monitoring damage build-up during SHI irradiation. While transmission electron microscopy (TEM) and atomic force microscopy (AFM) enable direct observation of ion tracks, RBS/c has been used in many studies for detailed research of ion track evolution vs. electronic stopping power [3,7,27,28]. Damage kinetics observed by RBS/c provides additional information about the mechanism of ion track formation, i.e., whether multiple SHI hits are necessary for amorphization of the material or if a single SHI impact process suffices [28,29,30,31]. Detailed monitoring of damage build-up within the material during SHI irradiation is particularly of interest close to the ion track formation threshold, where deviations from simple overlap track models are expected [29]. However, such detailed studies require irradiation and RBS/c measurements on a large number of samples demanding a large amount of beamtime. Recently, a solution to this problem was implemented in the establishment of experimental set-ups for in situ measurements using different analytical techniques like IL [32,33,34,35], X-ray diffraction [36], AFM [37], and Raman Spectroscopy [38,39].

These developments touch upon another important aspect of in situ measurements, allowing samples to be investigated without breaking the vacuum. For example, adsorbed water layers are frequently observed during AFM studies of ion tracks on CaF_2_ surfaces [40,41], and clean Si surfaces are prone to very fast oxidation [42,43]. Furthermore, 2D materials like graphene and MoS_2_ can become chemically reactive by the ion-introduced defects, especially at the location of the ion impact or on the edge of the ion-introduced pores [19,21,44]. By taking the irradiated samples out of the vacuum, follow-up measurements under ambient conditions can be affected, and for this reason AFM measurements should be preferably done in vacuum [37]. Since Raman spectroscopy is a very powerful analytical technique that can probe directly into damage kinetics of 2D materials [19,21,45,46], the in situ Raman spectroscopy set-ups that have been commissioned over the last few years [38,39] should be very interesting for ion-irradiation studies of 2D materials.

Here, we present three approaches for in situ measurements of the ion tracks using IBA techniques available at the Ruđer Bošković Institute (RBI) accelerator facility in Zagreb [47]. In the first case, we describe a newly built dual-beam chamber where material modifications can be induced by a SHI beam delivered from the 6 MV EN Tandem Van de Graaff accelerator, while monitoring of the damage kinetics is completed simultaneously by RBS/c using a probing beam from the 1 MV Tandetron accelerator. Feasibility of this approach is shown on radiation sensitive CaF_2_, and demonstration of the in situ experiment is shown on quartz SiO_2_. In the second example, we show how time-of-flight elastic recoil detection analysis (ToF-ERDA) can be utilized for monitoring the surface stoichiometry during grazing incidence SHI irradiation that results in very long ion tracks on the material surface. Previously, we have shown that ion track formation in GaN [18] and TiO_2_ [20] is accompanied by a preferential loss of nitrogen and oxygen, respectively. However, ion track formation in CaF_2_ does not result in changes of the surface stoichiometry under the same irradiation conditions [10]. Here, we present results of similar ToF-ERDA measurements made on other track forming materials, namely quartz SiO_2_, amorphous SiO_2_, SrTiO_3_, and muscovite mica. The third case is related to the open question about the origin of the velocity effect, i.e., what is the mechanism behind an increasing damage cross section (ion track size) for low velocity SHI irradiation in insulators [7,9,10]? Szenes proposed that the damage cross section increase is related to the activation of the Coulomb explosion mechanism due to the high ionization of the matter along the ion trajectory for slower ions [9]. The in situ high resolution particle induced X-ray emission (HR-PIXE) measurements during ion irradiation and the ion track formation demonstrated here could be a useful tool to evaluate this effect. To test the suitability of our HR-PIXE set-up for experimental testing of Szenes’s interpretation, we present the analysis of in situ HR-PIXE measurements of Si K X-ray spectra acquired during irradiation of amorphous SiO_2_.

## 2. Experiments and Results

### 2.1. Materials

Epi-ready single crystal quartz SiO_2_ (0001) and SrTiO_3_ (100) samples as well as thin amorphous SiO_2_ films (200 nm) grown on a Si wafer were purchased from Crystec (Berlin, Germany). The CaF_2_ (100) sample was cleaved prior to irradiation from the single crystal block purchased from Korth Kristalle (Kiel, Germany). Muscovite mica samples were obtained from 2SPI, and their surfaces were freshly cleaved prior to irradiation. Finally, a 500 nm thin amorphous SiO_2_ layer was grown on an Al_2_O_3_ (1102) substrate by magnetron sputter deposition at the RBI [13,14].

### 2.2. Monitoring Ion Track Production by In Situ RBS/c

**Figure 1:**
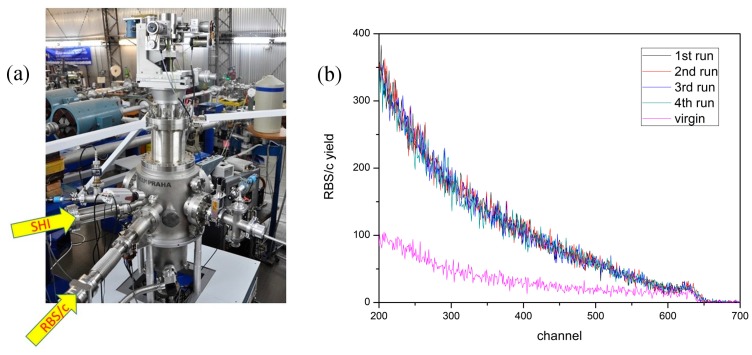
(**a**) Dual beam vacuum chamber for simultaneous swift heavy ions (SHI) irradiation and in situ Rutherford backscattering spectrometry in channeling mode (RBS/c) measurements; (**b**) Four successive RBS/c spectra obtained from an irradiated CaF_2_ sample (23 MeV I, fluence 3 × 10^12^ cm^−2^). Spectrum from the virgin sample shown for comparison.

In the first study, the experiment was performed in the new dual beam chamber equipped with a 6-axis goniometer (Figure 1a) using 5 MeV Si, 4 MeV C, 2 MeV Li and 1 MeV p beams delivered from the 1 MV Tandetron accelerator. For the RBS/c measurement, the probing ion beam had a 1 mm beam spot in diameter, and the current was kept at around 1 nA. To detect backscattered ions, a silicon surface barrier (SSB) detector was positioned at 160° with respect to the probing beam direction. The angular scan maps (tilt, azimuth) were acquired for target alignment.

For in situ RBS/c analysis, it is important that repeated RBS/c measurements at the same spot do not introduce additional damage to the material under investigation. Therefore, we performed test RBS/c measurements using a 2 MeV Li probing beam on the ion radiation sensitive CaF_2_ crystal, which contained defects introduced previously by 23 MeV I [10]. This verified that the RBS/c beam does not introduce additional defects after prolonged exposure, and that RBS/c spectra from irradiated samples (containing disorder) can, therefore, be reliably acquired even after multiple probing beam exposures, as shown in Figure 1b.

While more energetic ion beams from the 6 MV EN Tandem Van de Graaff accelerator can be inserted into the dual beam chamber as well, for the present study of damage kinetics in quartz SiO_2_, the 5 MeV Si and 4 MeV C beams delivered by the 1 MV Tandetron accelerator were found sufficient. Irradiations were done on two 1 × 1 cm^2^ single crystal quartz SiO_2_ (0001) samples that were irradiated on several positions by different fluences of 5 MeV Si and 4 MeV C ions, having electronic stopping powers of *S_e_* = 3.2 keV/nm and *S_e_* = 1.55 keV/nm, respectively [23]. For these two ion beams, nuclear stopping powers were *S_n_* = 0.028 keV/nm and *S_n_* = 0.003 keV/nm, respectively [23]. Irradiations by the 5 MeV Si beam were performed at 6° off normal (i.e., random), while irradiations by the 4 MeV C beam were performed both in the channeling direction (on-axis) and 6° off normal. The RBS/c probing beam in both cases was 1 MeV p.

**Figure 2:**
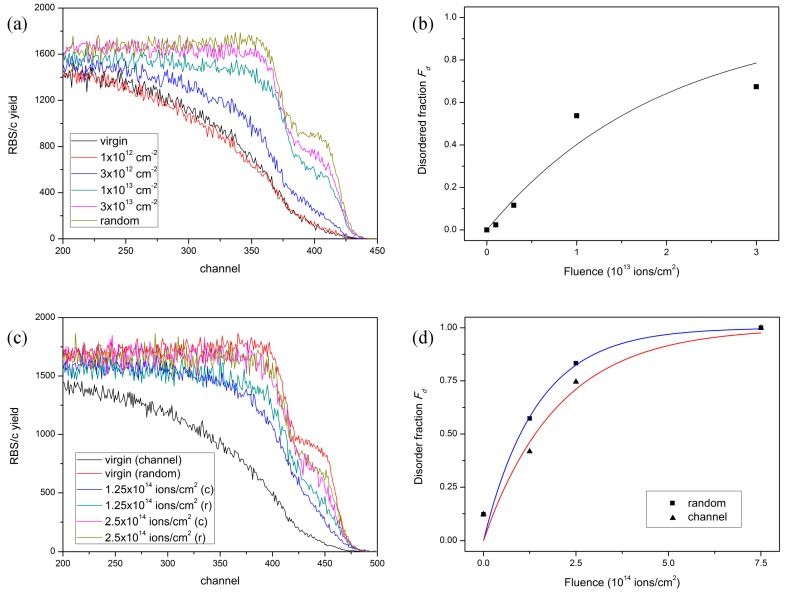
(**a**) RBS/c spectra from quartz SiO_2_ irradiated with different fluences of 5 MeV Si ions; (**b**) Analysis of RBS/c data yields ion track radius *R* = 1.3 ± 0.15 nm; (**c**) RBS/c spectra from quartz SiO_2_ irradiated with different fluences of 4 MeV C ions, both in channeling and random directions; (**d**) Analysis of RBS/c data yields damage cross sections of *σ_R_* = 0.7 ± 0.1 nm^2^ (blue line) and *σ_C_* = 0.5 ± 0.1 nm^2^ (red line) for the random and channeling irradiations, respectively.

For the analysis of the 5 MeV Si irradiated sample, the highest energy part of RBS/c spectra (Figure 2a) coming from backscattered protons on the Si sublattice was used to calculate the amount of disorder, i.e., the disordered fraction *F_d_*. Using the well-known procedure based on the surface approximation, the ion track radius *R* can be derived from Poisson’s law that describes the evolution of the disordered fraction *F_d_* with the applied SHI fluence [7,10,27]. As shown in Figure 2b, the RBS/c spectra provide evidence that gradual disordering of the quartz SiO_2_ sample takes place with increasing 5 MeV Si fluence, and for the highest applied fluence, almost complete amorphization takes place. From the observed damage kinetics, we evaluated the ion track radius to be *R* = 1.3 ± 0.15 nm, in agreement with previous works, where damage has been found below 3 keV/nm [27,28].

The analysis of the RBS/c spectra from the 4 MeV C irradiated sample follows a slightly different procedure [48], and spectrum integration between channels 425–475 (Figure 2c) was taken as a measure of disorder. This approach was found adequate for the analysis of damage occurring below threshold for ion track formation, and the electronic stopping power of the 4 MeV C beam is below the reported threshold of 2 keV/nm [28]. Clearly, large fluences are needed to introduce observable damage, but this is not compelling evidence to assign origin of this damage to nuclear stopping power [30,48]. For example, it is generally accepted that electronic excitations in quartz can result in exciton self-trapping that can lead to structural changes upon their annihilation [1,4,5]. While the process causing damage is not clear (nuclear stopping power or self-trapped exciton mechanism), we show in Figure 2c,d that the presented in situ RBS/c set-up enables detailed measurements of the channeling and near-channeling effects influencing ion track formation [22,49,50].

### 2.3. Evaluating Ion Track Stoichiometry by In Situ ToF-ERDA

For the ToF-ERDA study, SrTiO_3_, quartz SiO_2_, amorphous SiO_2_, and muscovite mica samples were used. All the surfaces were found suitable for the AFM analysis. Grazing incidence irradiation was applied using a 23 MeV I beam delivered by the 6 MV EN Tandem Van de Graaff accelerator, and ToF-ERDA was carried out on the dedicated chamber described in detail elsewhere [51,52]. The ToF-ERDA measurements were performed at 1° grazing incidence angle with respect to the sample surface, with the spectrometer positioned at the angle of 37.5° towards the beam direction. All data was collected in the “list mode” and offline replay/analysis with sections was performed using the Potku software package [53]. Afterwards, ion tracks on the surfaces were analyzed using tapping mode AFM. The AFM measurements were performed under ambient conditions using a Dimension 3100 AFM and Nanosensors NCHR cantilevers at Universität Duisburg-Essen. Images were analyzed using the WSxM code [54].

**Figure 3:**
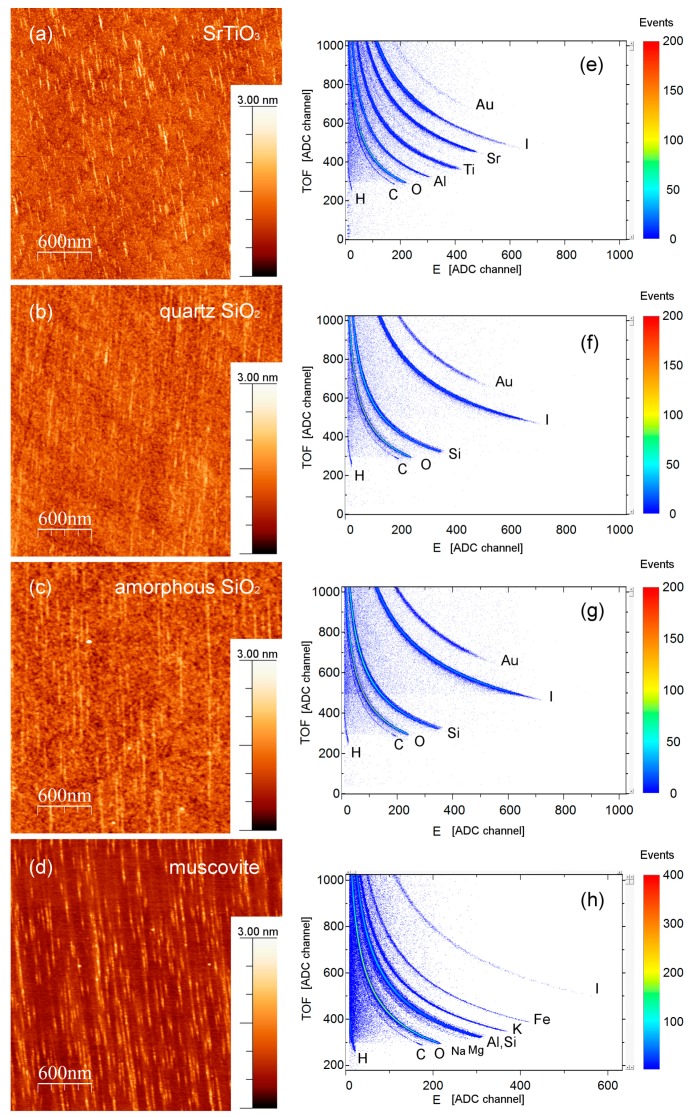
Ion tracks on the material´s surfaces produced by 23 MeV I ions under a grazing incidence angle of 1°, observed by atomic force microscopy (AFM): (**a**) SrTiO_3_; (**b**) quartz SiO_2_; (**c**) amorphous SiO_2_; (**d**) muscovite mica. In all cases, the applied ion fluence matches well the observed ion track density. Corresponding grazing incidence time-of-flight elastic recoil detection analysis (ToF-ERDA) spectra obtained by the same 23 MeV I beam are shown in panels (**e**–**h**). Detection of iodine is from the primary ion beam, while detected Al and Au are from the gold coated aluminum sample holder.

Ion tracks were formed on all investigated surfaces after exposure to the 23 MeV I beam under 1° grazing incidence angle (Figure 3). According to the SRIM code [23], electronic stopping powers were above track forming thresholds, i.e., 5.2 keV/nm (amorphous SiO_2_), 6.3 keV/nm (quartz SiO_2_), 6.5 keV/nm (muscovite mica), and 9 keV/nm (SrTiO_3_). The observed surface tracks were aligned along the direction of the incident beam and as such were easily seen in the AFM images, as Figure 3 shows. For all investigated samples, the density of the ion tracks agrees well with the applied ion fluence, i.e., efficiency of the ion track formation is close to 1.

Figure 3 also shows ToF-ERDA spectra collected during exposure to the same SHI beam and at the same 1° grazing incidence angle. The applied fluence necessary for acquiring ToF-ERDA spectra was typically 10^12^ ions/cm^2^, resulting in multiple ion track overlap. In the case of quartz SiO_2_, amorphous SiO_2_ and muscovite mica measurements, stoichiometry change was monitored for the first ~10–12 nm of the sample (depth resolution of ~2.5–3 nm) by replying the measured spectra. The same procedure was performed for the SrTiO_3_ sample, but this time, due to the different sample density, stoichiometry change was tracked in the first ~3.8 nm of the sample with depth resolution of ~1.4 nm. It was found that for all investigated materials, surface elemental composition (stoichiometry) remained unchanged. Therefore, we conclude that ion track formation in investigated materials is not accompanied by the preferential loss of any element. Calculated “surface” atomic concentrations, for the all samples, are reported in the Table 1.

### 2.4. Observing First Stages of Ion Track Formation Using In Situ HR-PIXE

In this study, we used a 500 nm thin amorphous SiO_2_ layer grown on a Al_2_O_3_ substrate. The HR-PIXE spectrometer [55,56] was mounted on the ion microprobe set-up. The sample was irradiated using 12 MeV O^4+^ and 6 MeV O^3+^ ion beams from the 6 MV EN Tandem accelerator. The aim of this experiment was to study the *K*- and *L*-shell ionization probabilities of the silicon atoms after passage of these ion beams, by observing the silicon *KαL^N^* X-ray emission, where *N* is the number of spectator holes in the *L*-shell during the *K-L*_3_ and *K-L*_2_ X-ray transitions (i.e., *Kα*_1_ and *Kα*_2_).

**Figure 4:**
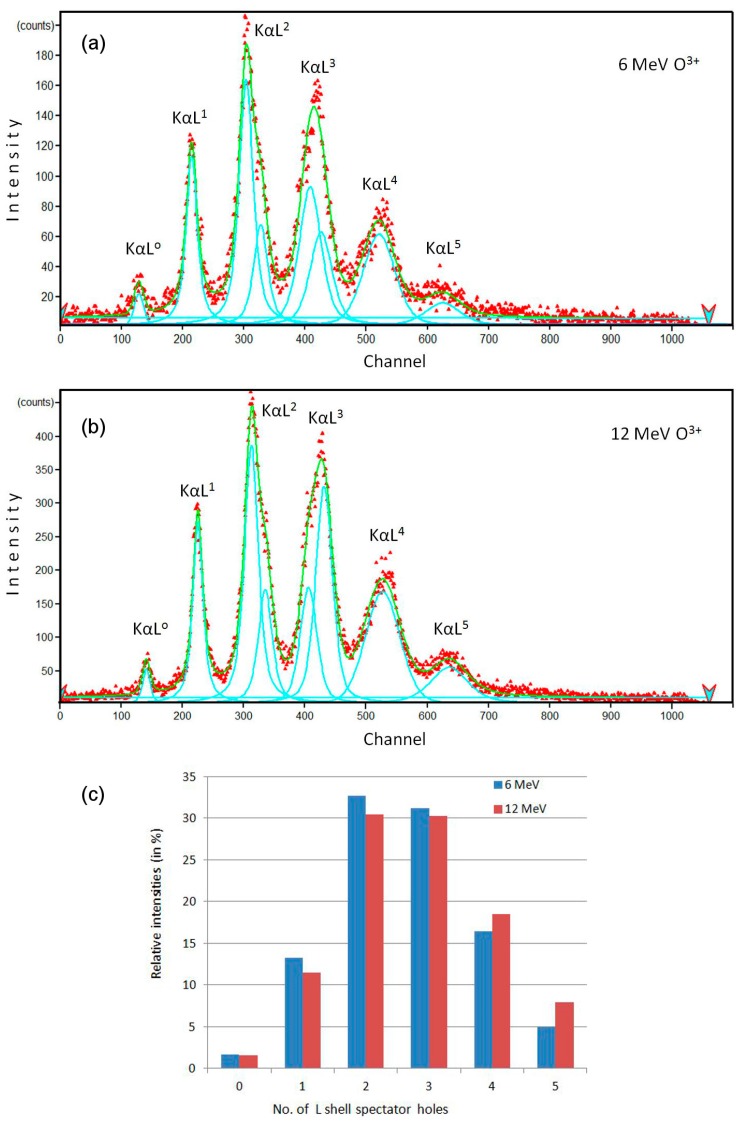
High resolution particle induced X-ray emission (HR-PIXE) spectra of Si *KαL^N^* X-rays from thin a-SiO_2_ film irradiated by (**a**) 6 MeV O^3+^ and (**b**) 12 MeV O^4+^ ion beam; (**c**) Relative intensities of *KαL^N^* X-rays depending on the number of spectator holes in the *L*-shell.

In the acquired spectra (Figure 4a,b), the observed lines were fitted to Gaussian peaks using the WxEWA (V0.29a-Alpha8) software package [57]. As shown in the Table 2 and Figure 4c, the observed differences are minor, suggesting that the populations of electrons and holes in the inner shells of the Si atoms are quite similar for these two ion irradiation conditions. Such experimental observation can help to shed light on the proposed mechanism of Coulomb explosion [9] in the formation of ion tracks, since 6 MeV O^3+^ ions produce ion tracks in a-SiO_2_, but 12 MeV O^4+^ ions should not produce ion tracks in a-SiO_2_ due to the velocity effect [14]. A more detailed analysis and further systematic measurements need to be done in order to arrive at definite conclusions. The results presented here show that high resolution measurements can be done reliably using our HR-PIXE set-up with a spectral resolution of ~1 eV, which is sufficient for quantitative analysis (Table 2). This analysis can further proceed in the same manner as reported in references [58,59], when *L*-shell and even *M*-shell vacancy distributions can be obtained. Compared to the abovementioned work where a low-density SiO_2_ aerogel was used for spatially resolved X-ray measurements, X-rays detected from thin SiO_2_ film can give exact information about the primary stages of ion track formation for specific irradiation conditions (ion type, energy, and charge state) relevant to ion track formation.

## 3. Discussion

Increased interest for in situ ion track measurements stems from the possibility of acquiring numerous data points from a single sample during a single irradiation run. This approach can save a lot of valuable beamtime and provide large amount of data necessary for detailed investigations of damage kinetics [28,29,30,31]. Besides our on-going efforts to utilize grazing incidence ToF-ERDA for in situ elemental analysis of ion tracks [10,18,20], here, we introduced in situ RBS/c for monitoring damage build-up due to the production of ion tracks. This is especially important for ion track studies done close to the threshold for ion track formation. Below the threshold, subthreshold damage arising from secondary damage mechanisms (for example the mechanism involving excitons in the case of TiO_2_ [31]) can be identified by the deviation of the experimental data from Poisson’s law. Similarly, above threshold behavior described by the Avrami equation can also indicate discontinuities within small ion tracks [29]. The experimental data we have shown in Figure 2 are consistent with results published earlier [28], but a deviation from the Poisson law due to the proximity to the ion track formation threshold can be suspected. While a more detailed picture of the damage kinetics is probably not possible to achieve using proton beams, the use of the 2 MeV Li beams requires significantly longer time for the collection of the RBS/c spectra. Therefore, measurements using He beams (to be available after the next upgrade of the Tandetron ion source system) remain to be performed in our laboratory.

Although IBA techniques are generally considered to be non-destructive, clearly one has to be careful about the possible damage introduced by the probing RBS/c beam. Care has to be taken particularly for He and Li ion beams that have tenfold larger stopping powers than proton beams. Surprisingly, we have found that even for a sensitive material like CaF_2_, the damage of the probing 2 MeV Li beam was below the detection limit, and RBS/c measurements repeated on the same spot on the sample surface yield the same result. This is in stark contrast to the great sensitivity of this material to the e-beam induced damage and related difficulties during the TEM observations [10,60,61]. Although the RBS/c probing beam dissipates energy within CaF_2_ via electronic excitations, secondary electrons generated this way have two orders of magnitude lower energy than electrons used in the TEM. Therefore, damage to the material via electronic excitations is suppressed and the use of RBS/c data is clearly a better choice for thermal spike studies than TEM data [10].

In situ ToF-ERDA measurements have been used before to monitor stoichiometry changes during SHI irradiation [62,63,64]. The results presented here and in our previous publications [10,18,20] demonstrate the feasibility of in situ ToF-ERDA performed under the 1° grazing incidence angle, thus enabling monitoring of stoichiometry changes under conditions for surface ion track formation. With the exception of GaN that obviously decomposes during the thermal spike produced by the ion impact [18], and a still unclear mechanism of preferential oxygen loss from TiO_2_ [20], all other investigated materials show stable stoichiometry during SHI irradiation with the similar stopping powers. Furthermore, it would be of interest to compare these findings with results of the sputtering experiments [65]. 

HR-PIXE takes in situ ion track analysis one step further. This technique is not limited to merely post-mortem analysis of ion tracks, where properties of ion tracks are deduced indirectly via damage kinetics or stoichiometry changes. HR-PIXE offers direct insight into the earliest processes occurring during the ion track formation [58,59]. Ionoluminescence can also provide similar information, but the timescale of the observed processes is on a much slower sub-nanosecond scale [32,33]. Results presented here demonstrate that the experimental capabilities of our HR-PIXE set-up are sufficient for investigating the early stages of ion track formation. Although the complete analysis leading to ionization probabilities of atoms within the ion track is not facile [58,59], results will be highly valuable and relevant for our understanding of ion track formation processes.

## 4. Conclusions

For the first time, we have successfully demonstrated in situ RBS/c measurements of ion tracks. The dual beam set-up presented here will enable precise monitoring of the damage build-up within a given material during SHI irradiation. Surprisingly, RBS/c, using heavier ions like lithium instead of protons, does not introduce additional damage to sensitive materials like CaF_2_. Therefore, multiple RBS/c measurements on the same spot yield reliable results that are necessary for in situ RBS/c. This approach is especially important for studies close to the ion track formation threshold, where deviations from simple overlap track models are expected. To accomplish this, a substantial amount of experimental data is needed for reliable analysis, and this novel approach could provide an adequate solution to this challenge.

We have also presented new experimental data on in situ grazing incidence ToF-ERDA measurements. Using this technique, the elemental composition of the surface can be monitored under the conditions when surface ion tracks are produced. Any change in the stoichiometry of the surface provides evidence about the elemental composition of the ion tracks. In the present study, we found that the surface stoichiometry of the investigated materials (SrTiO_3_, muscovite mica, quartz SiO_2_, and a-SiO_2_) remains unchanged. The HR-PIXE was the third IBA technique we presented for analysis of ion tracks. We demonstrated that our HR-PIXE set-up at the ion microprobe has sufficient spectral resolution for the detailed analysis necessary for measuring ionization probabilities of inner shell electrons in silicon atoms.

## Figures and Tables

**Table 1:** Calculated “surface” atomic concentrations for quartz SiO_2_, amorphous SiO_2_, muscovite mica, and SrTiO_3_.

Sample	Atomic Concentration (%)	Density (g/cm^3^)	Analyzed Depth (nm)	Depth Resolution (nm)
quartz SiO_2_	O: (67 ± 4) Si: (32 ± 2)	2.65	~10	~2.5
amorphous SiO_2_	O: (67 ± 4) Si: (32 ± 2)	2.2	~12	~3
muscovite mica	H: (2.6 ± 0.2) C: (1.6 ± 0.1) O: (58 ± 3) Na: (0.67 ± 0.06) Mg: (0.86 ± 0.07) Al: (16 ± 1) Si: (14 ± 1) K: (4.6 ± 0.3) Fe: (1.3 ± 0.1)	2.82	~10	~2.5
SrTiO_3_	O: (60 ± 3) Ti: (21 ± 1) Sr: (19 ± 2)	5.1	~3.8	~1.4

**Table 2:** Relative intensities of *KαL^N^* X-rays for 6 MeV O^3+^ and 12 MeV O^4+^ irradiation.

Ion Beam	L0	L1	L2	L3	L4	L5
6 MeV O^3+^	1.68 ± 0.12	13.21 ± 0.41	32.65 ± 0.72	31.17 ± 0.75	16.4 ± 0.3	4.90 ± 0.29
12 MeV O^4+^	1.56 ± 0.11	11.44 ± 0.27	30.41 ± 0.56	30.26 ± 0.39	18.45 ± 0.29	7.89 ± 0.19

## Supplementary Materials

The following are available online at www.mdpi.com/1996-1944/10/9/1041/s1, experimental data from RBS/c, AFM, ToF-ERDA, and HR-PIXE.
